# Ubiquitin‐Proteasome System in Periodontitis: Mechanisms and Clinical Implications

**DOI:** 10.1111/cpr.13781

**Published:** 2024-12-03

**Authors:** Yilin Ma, Ruiwei Jia, Shuhong Chen, Jun Ma, Lei Yin, Xingbei Pan, Yunuo He, Tong Wu, Zheyu Zhao, Lulu Ma, Shengzhuang Wu, Huining Wang, Guang Liang, Shengbin Huang, Xiaoyu Sun

**Affiliations:** ^1^ Institute of Stomatology, School and Hospital of Stomatology Wenzhou Medical University Wenzhou China; ^2^ Institute of Stomatology, School and Hospital of Stomatology Hangzhou Medical College Zhejiang Hangzhou China; ^3^ Department of Periodontics, School and Hospital of Stomatology Wenzhou Medical University Wenzhou China; ^4^ Department of Prosthodontics, School and Hospital of Stomatology Wenzhou Medical University Wenzhou China

**Keywords:** bone metabolism, deubiquitinating enzymes, E3 ubiquitin ligases, inflammation, periodontitis, ubiquitin‐proteasome system

## Abstract

The progression of periodontitis, a bacteria‐driven inflammatory and bone‐destructive disease, involves myriad cellular and molecular mechanisms. Protein regulation significantly influences the pathogenesis and management of periodontitis. However, research regarding its regulatory role in periodontitis remains relatively limited. The ubiquitin‐proteasome system (UPS), which mainly involves ubiquitination by E3 ubiquitin ligases (E3s) and deubiquitination by deubiquitinating enzymes (DUBs), is the primary intracellular and non‐lysosomal mechanism of protein degradation. Recent studies have provided compelling evidence to support the involvement of UPS in periodontitis progression. Increasing evidence indicated that E3s, such as CUL3, Nedd4‐2, Synoviolin, FBXL19, PDLIM2, TRIMs and TRAFs, modulate inflammatory responses and bone resorption in periodontitis through multiple classical signalling pathways, including NLRP3, GSDMD, NF‐κB, Wnt/β‐catenin and Nrf2. Meanwhile, DUBs, including OTUD1, A20, CYLD, UCH‐L1 and USPs, also broadly modulate periodontitis progression by regulating signalling pathways such as NF‐κB, Wnt/β‐catenin, NLRP3, and BMP2. Therefore, the modulation of E3s and DUBs has proven to be an effective therapy against periodontitis. This review provides a comprehensive overview of the regulatory role of ubiquitinating and deubiquitinating enzymes in periodontitis progression and the underlying mechanisms. Finally, we summarise several chemical and genetic methods that regulate UPS enzymes and pave the way for the development of targeted therapies for periodontitis.

## Graphical abstract

A literature review outlines the regulatory role of E3s and DUBs in inflammatory response and bone resorption involved in periodontitis.

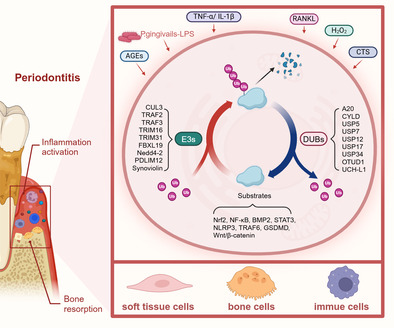

## 1 Introduction

Periodontitis, a leading cause of tooth loss in adults, is a common inflammatory disease that severely damages tooth‐supportive tissues and impacts approximately 796 million individuals globally [1, 2]. Its strong association with systemic diseases, such as diabetes mellitus, cardiovascular disease and obesity, amplifies its socioeconomic burden [3]. Moreover, the absence of early diagnostic markers and effective therapeutic targets highlights the urgent need for a comprehensive understanding of the pathological mechanisms underlying periodontitis [4].

Protein regulation significantly influences the pathogenesis and management of periodontitis [5, 6]. Post‐translational modifications (PTMs) are essential mechanisms of protein regulation that involve amino acid side chain modifications of proteins after biosynthesis [7]. PTMs have significant effects on protein structure and function. Disruption of PTMs leads to the malfunction of vital biological processes and, consequently, to various diseases [8]. Various inflammatory and bone metabolism‐related factors, such as nuclear factor‐kappa B (NF‐κB), gasdermin D (GSDMD), and receptor activators of NF‐κB ligand (RANKL), are regulated by PTMs [8, 9]. Elucidating the potential mechanisms through which protein regulation influences periodontitis progression will provide vital insights into the pathophysiology of periodontitis.

The ubiquitin‐proteasome system (UPS) is the principal intracellular and non‐lysosomal protein degradation mechanism that is responsible for the breakdown of over 80% of cellular proteins. Dysregulation of the UPS is linked to the development of numerous diseases, including neurodegenerative diseases, cancer and metabolic disorders [10]. E3 ubiquitin ligases (E3s) and deubiquitinating enzymes (DUBs) are key components of the UPS. E3s recognise specific substrates for modification by facilitating the transfer of ubiquitin (Ub) from E2 to substrate proteins [11]. DUBs reverse ubiquitination by removing Ub from ubiquitinated proteins [12]. Recent research highlighted the significant involvement of UPS in the pathogenesis of periodontitis. Research findings from animal and human studies have revealed altered levels of E3s and DUBs within periodontal tissues during periodontitis progression [13, 14]. Moreover, E3s and DUBs influence the number and functionality of various cells in periodontal tissues and subsequently affect inflammatory and immune responses and bone metabolism in periodontitis [15]. Numerous studies have revealed that targeted regulation of E3s and DUBs alleviated periodontitis progression, suggesting that these enzymes potentially serve as therapeutic targets for periodontitis [16, 17].

Various cell types, including periodontal ligament stem cells (PDLSCs), periodontal ligament cells (PDLCs) and gingival fibroblasts (GFs), influence periodontal tissues through metabolic functions and regenerative capacity. PDLSCs, the main progenitor cells in periodontal tissue, are identified as mesenchymal stem cells and the ideal seed cells for periodontal tissue regeneration [18]. PDLCs are the major components of the periodontal ligament [19], while GFs are the most abundant cells in the gingival connective tissue that maintain tissue structure and integrity [20]. Additionally, cells related to bone metabolism, such as osteoblasts, osteoclasts and osteocytes, play a significant role in periodontitis progression [21]. More notably, immune cells, including macrophages, neutrophils, peripheral blood mononuclear cells (PBMCs) and T helper cells 17 (Th17), play vital roles in the development of periodontitis by defending against periodontal pathogens and mediating inflammatory responses [22].

We sought to provide a review of recent research on the involvement of E3s and DUBs in periodontitis and the underlying mechanisms. This review also sought to establish a foundation for novel therapeutic strategies against periodontitis.

## 2 Ups

**FIGURE 1:**
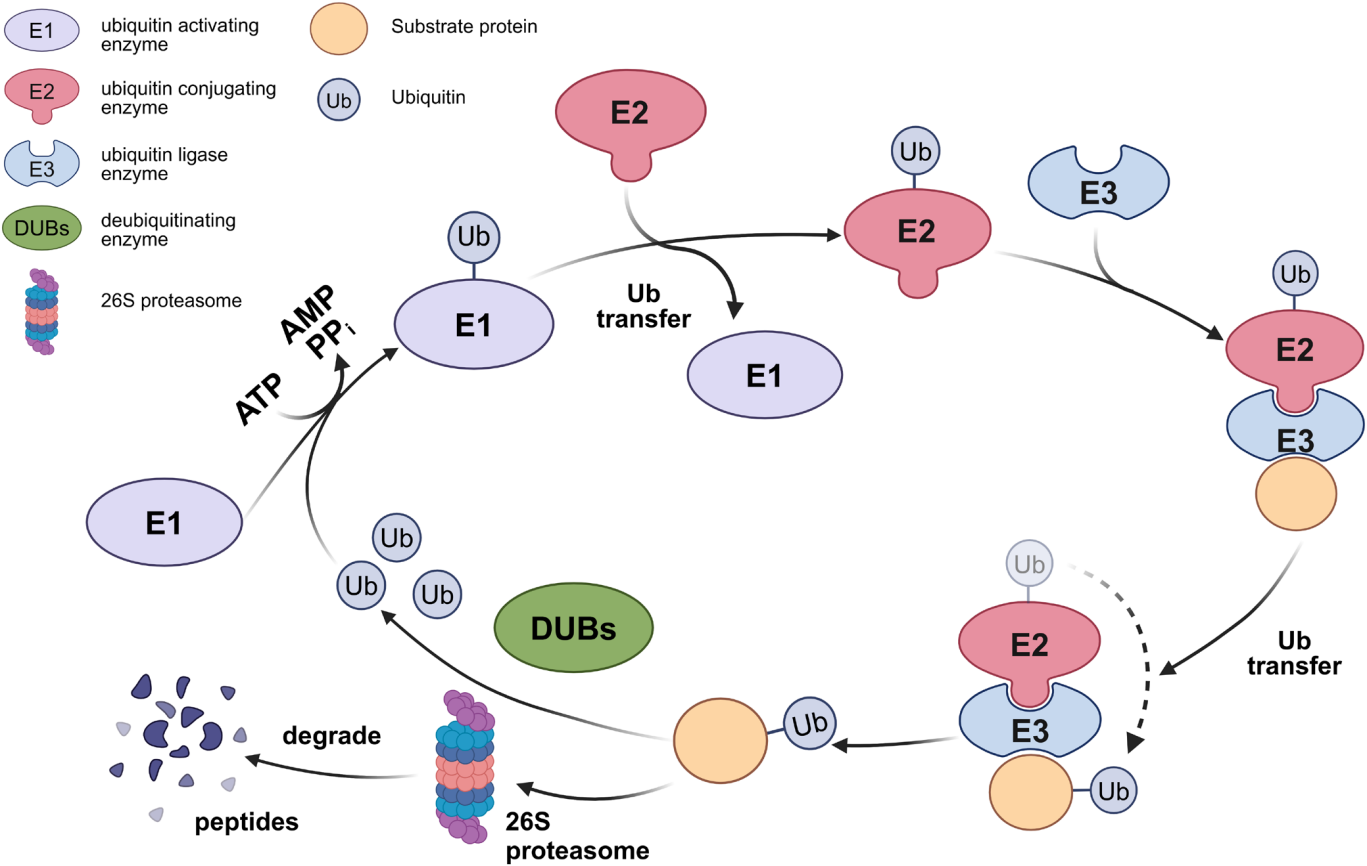
Ubiquitination and deubiquitination processes within UPS. E1 activates Ub by forming a Ub‐AMP adenylate and transfers Ub to E2. E3 ligase facilitates the transfer of Ub to substrate proteins marked for degradation by the 26S proteasome. DUBs remove Ub chains, regulating degradation and recycling ubiquitin molecules.

UPS is the primary intracellular and non‐lysosomal protein degradation system related to protein quality control and homeostasis, and involves a balance between ubiquitination and deubiquitination (Figure 1) [23].

### 2.1 Ubiquitination and E3s

Ub is a small and highly conserved protein with a molecular weight of 8.5 kDa. Protein ubiquitination involves the attachment of Ub to target proteins, resulting in the formation of Ub chains that modify substrate proteins [24]. Ubiquitination is pivotal in regulating various cellular functions, including protein degradation, designation of protein subcellular localization and induction of protein–protein interactions [25]. Ubiquitination modifications are mediated by the E1‐E2‐E3 cascade reaction. E1 initiates the activation of Ub by triggering a reaction between the C‐terminal carboxylate of Ub and adenosine 5′‐triphosphate (ATP), forming Ub‐AMP adenylate [26]. The activated Ub molecule is subsequently captured by the catalytic Cys residues of E1. Activated Ub is then converted to E2 via thioester exchange reactions. Ultimately, E3 facilitates the transfer of Ub from E2 to substrate proteins. Subsequently, proteins tagged with Ub are targeted for degradation by the 26S proteasome [10].

E1s and E2s possess highly conserved sequences and structures, whereas E3s exhibit high structural variability. E3s are categorised into four types based on their structural and functional distinctions: homologous to the really interesting new gene (RING), U‐box, e6‐associated protein carboxyl‐terminus (HECT) and cullin‐RING ligases (CRLs). RING, U‐box and HECT are single‐subunit E3s, whereas CRL‐type E3s are multi‐subunit E3s. RING‐type E3s facilitate the transfer of activated Ub from an E2 conjugate to a protein substrate. U‐box‐type E3s interact with E2s through the U‐box structural domain, enabling U‐box‐type E3s to transfer Ub directly from E2s to target proteins for degradation. HECT‐type E3s transfer Ub directly to the substrate and simultaneously coordinate transfer with the E2 conjugating enzyme. CRLs are the largest family of E3s in mammalian cells, which manage substrate‐specific recognition and the process of ubiquitination modification and degradation. Therefore, CRLs are critical regulators of various cellular functions, ensuring the normal functioning of biological processes [27, 28].

Ubiquitination modifications are categorised into nine types based on the linkage sites of Ub molecules, including k6, k11, k27, k29, k33, k48, k63, k76 and M1. Among these types, k48‐linked and k63‐linked ubiquitination have been the subject of extensive research. K48‐linked ubiquitination primarily labels proteins for recognition and degradation by the 26S proteasome [29]. K63‐linked polyubiquitin chains, however, modulate the activity, interaction, or intracellular trafficking of tagged proteins, participating in various biological procedures [30, 31].

Ubiquitination is the predominant modification that regulates protein degradation and significantly modulates cell growth, proliferation and survival. Dysregulation of ubiquitination leads to a variety of diseases, such as cancer, metabolic syndrome and neurodegenerative disorders [24]. Given their diverse structures, E3s can be modified in various ways and potentially employed as therapeutic targets for the prevention of periodontitis [27].

### 2.2 Deubiquitination and DUBs

Ubiquitination is a reversible PTM that plays a dynamic role in protein degradation by adding Ub to substrate proteins. DUBs are specialised proteases that remove Ub from their substrates or cleave Ub chains for reverse ubiquitination. Approximately 100 human DUBs counteract the attachment of Ub signals by E3 ubiquitin ligases [32]. DUBs are categorised into two primary groups: metalloproteases and cysteine proteases. Most DUBs are cysteine proteases and are further divided into six subclasses: ubiquitin‐specific proteases (USPs), ubiquitin C‐terminal hydrolases (UCHs), ovarian tumour proteases (OTUs), machado–Josephin domain proteases (MJDs), the motif interacting with the ubiquitin‐containing DUB family (MINDY) and zinc finger with a UFM1‐specific peptidase domain protein/zinc finger containing Ub peptidase 1 (ZUFSP/ZUP1). The metalloproteinases comprise only the JAB1/MPN/Mov34 metalloenzyme (JAMMs) [33]. Among these families, USPs represent a subclass of DUBs that regulate protein levels and signalling pathways by removing Ub from targeted proteins [34]. The OTU family, the second‐largest DUB subfamily, possesses a core structural domain with three catalytically active sites (Cys, His and Asp) that control protein degradation, DNA repair and inflammatory responses [35]. DUBs are involved in a wide range of cellular physiological processes such as apoptosis, autophagy and immune responses [36]. Studies have highlighted the role of DUBs in regulating inflammatory responses and bone resorption, thereby influencing periodontitis progression [37].

Currently, the regulatory role of the UPS in the progression of periodontitis and its underlying mechanisms remain unclear. A deeper understanding of the UPS pathogenic pathways is integral to identifying potential therapeutic targets for periodontitis [38]. This review highlights the vital role of the UPS in the metabolism of periodontitis, explores potential avenues for future research and highlights innovative therapeutic strategies for periodontitis.

## 3 Role Of E3S in Periodontitis

**FIGURE 2:**
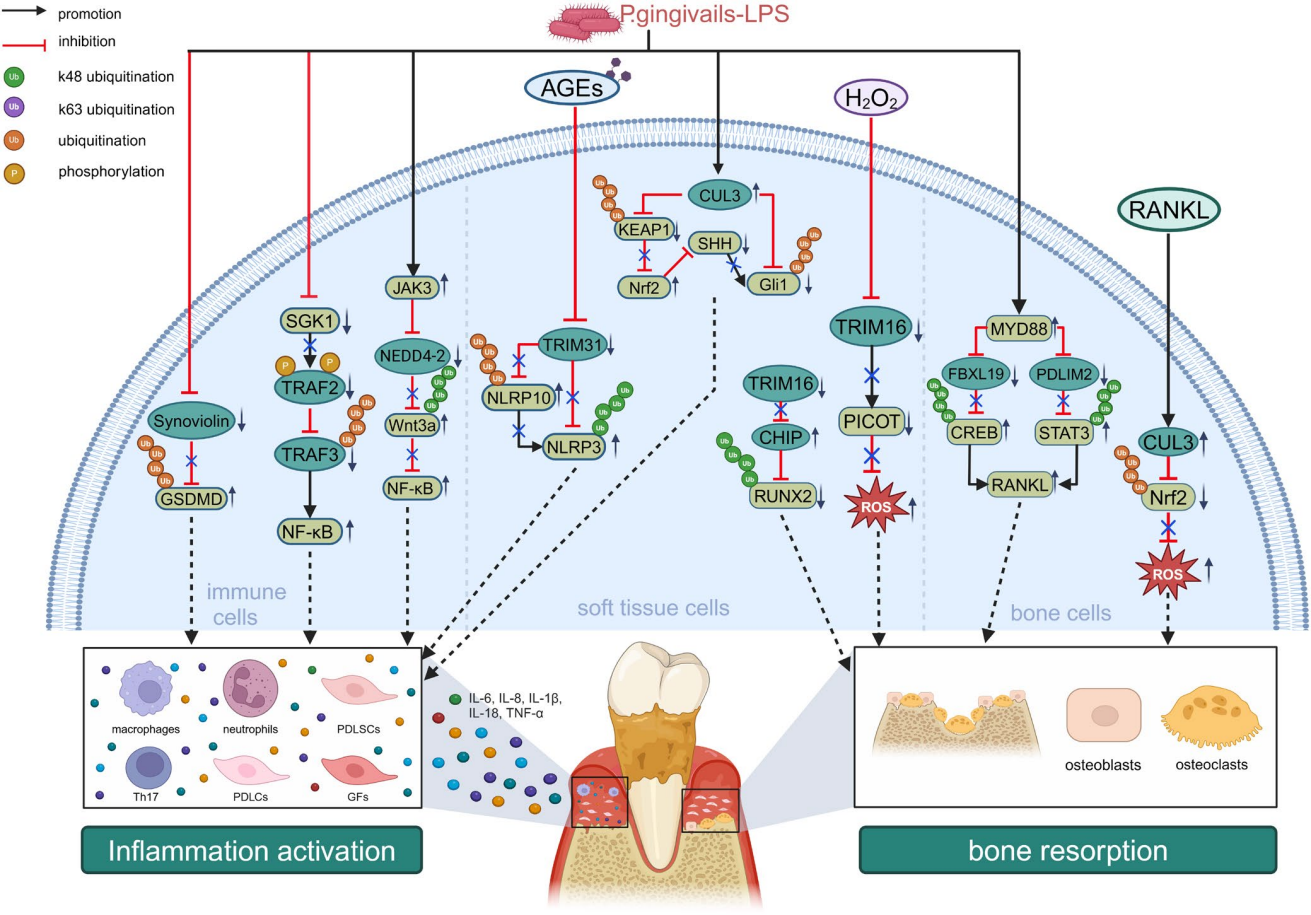
Effect of E3s on periodontal inflammation and bone resorption. CUL3 induced inflammation through ubiquitinating Gli1 and Keap1 upon P.g‐LPS stimulation. CUL3 induced bone destruction by promoting the ubiquitination of Nrf2 after RANKL stimulation. TRIM16 inhibited bone resorption by promoting the ubiquitination of PICOT and suppressing the ubiquitination of RUNX2 mediated by CHIP. TRIM31 inhibited inflammation by promoting ubiquitination of NLRP10 and NLRP3. PDLIM2 and FBXL19 downregulated RANKL and consequently inhibited bone resorption by promoting the ubiquitination of STAT3 and CREB, respectively. Nedd4‐2 suppressed inflammation through Wnt3a ubiquitination. TRAF2 induced inflammation through ubiquitinating TRAF3 degradation. Synoviolin inhibited inflammation via ubiquitinating GSDMD.

E3s, including Cullin3 (CUL3), Nedd4‐2, Synoviolin, FBXL19, PDLIM2, TRIMs and TRAFs have been identified as key players in modulating periodontitis. This section provides an overview of recent research concerning the involvement of E3s in regulating periodontitis‐related cells and the underlying mechanisms involved (Figure 2).

### 3.1 Role of E3s in Cells Related to Periodontal Soft Tissues

#### 3.1.1 Regulation of Inflammatory Responses and Osteoblastogenesis of PDLSCs by E3s

CUL3 is a scaffold protein that binds to the Bric‐a‐Brac‐Tramtrack‐Broad‐complex domain of substrate recognition adaptors and RING‐finger protein 1 (RBX1) to form an E3 ubiquitin ligase complex. Previous studies have shown that CUL3 recruited substrate‐specific adaptors to catalyse protein ubiquitylation and contributed to a multitude of cellular processes, such as cell division, differentiation and stress responses [39]. Lipopolysaccharides from *Porphyromonas gingivalis* (P.g‐LPS) are crucial pathogenic factors that lead to the onset and progression of periodontitis [40]. An earlier study highlighted that P.g‐LPS stimulation enhanced the protein expression of CUL3 by PDLSCs. Furthermore, the research revealed that overexpression of CUL3 weakened the differentiation and mineralisation capabilities of PDLSCs, and aggravated inflammation and apoptosis of PDLSCs. Mechanistically, CUL3 interacted with Kelch‐like ECH‐associated protein 1 (Keap1) to establish the Keap1‐CUL3‐E3 complex, which promoted the ubiquitination and proteasome‐dependent degradation of nuclear factor‐erythroid 2‐related factor 2 (Nrf2), thereby amplifying inflammatory responses. Furthermore, CUL3 downregulated the protein levels of glioma‐associated oncogene homologue 1 (Gli1), an important transcription factor within the SHH signalling pathway, thus facilitating the secretion of pro‐inflammatory cytokines and intensifying inflammatory responses [41]. Overall, CUL3 downregulated SHH/Gli1 and Nrf2, subsequently amplifying inflammatory responses and promoting the apoptosis of PDLSCs. Regarding the detrimental effect of CUL3 in exacerbating periodontitis, the application of DI‐1548 and DI‐1859, potent and selective inhibitors of CUL3, offer promising therapeutic strategies for treating periodontitis [42].

Tripartite motif‐containing proteins (TRIMs) represent the largest subfamily of single‐polypeptide RING E3s that share a conserved domain architecture [43]. Tripartite motif‐containing protein 16 (TRIM16), an integral member of the TRIM family, plays a key role in inhibiting oxidative stress and enhancing intracellular antioxidant capacity [44]. Clinical data revealed reduced TRIM16 protein levels in the gingival tissues of patients with periodontitis compared to healthy controls. Moreover, an inverse correlation was observed between TRIM16 protein expression and periodontal conditions such as plaque index, probing depth and probing bleeding index [45]. Furthermore, another study revealed that TRIM16 overexpression via plasmids enhanced the osteogenic differentiation of PDLSCs by upregulating runt‐related transcription factor 2 (RUNX2), an integral transcription factor for osteoblast differentiation. Consistently, TRIM16 inactivation by specific short‐hairpin RNA (shRNA) curtailed the osteogenic differentiation of PDLSCs. Furthermore, TRIM16 protected RUNX2 from proteasomal degradation. TRIM16 also decreased mRNA and protein levels of the carboxy terminus of the Hsp70 interacting protein (CHIP), a ubiquitin E3 ligase that induced RUNX2 degradation and inhibited osteoblast differentiation. More importantly, overexpression of CHIP attenuated the osteogenic capacity of TRIM16‐overexpressing PDLSCs by diminishing RUNX2 protein levels. Taken together, TRIM16 stabilised RUNX2 protein levels by decreasing CHIP‐mediated k48‐linked ubiquitination‐independent degradation of RUNX2, thereby fostering the osteogenic differentiation of PDLSCs [46].

Numerous studies have identified oxidative stress as the major pathological mechanism underlying periodontitis. Antioxidant therapy has been proved to reduce oxidative damage and alleviate alveolar bone loss in periodontitis [47]. Exposure of PDLSCs to hydrogen peroxide (H_2_O_2_) is a widely used oxidative damage model for periodontitis [48]. One study revealed that TRIM16 mitigated H_2_O_2_‐induced oxidative damage in PDLSCs by enhancing antioxidant capacity and reducing the amount of intracellular reactive oxygen species (ROS) and reactive nitrogen species. Mechanistically, TRIM16 overexpression activated protein kinase C‐interacting cousin of thioredoxin (PICOT), a crucial antioxidant enzyme that protected PDLSCs from oxidative damage. Subsequent investigations demonstrated that the recovery of cell viability induced by TRIM16 overexpression was abolished by PICOT knockdown via shRNA. Accordingly, TRIM16 overexpression alleviated periodontitis by promoting osteogenic differentiation of PDLSCs and inhibiting oxidative damage [49]. Therefore, activation of TRIM16 may represent a promising strategy for the prevention and treatment of periodontitis.

#### 3.1.2 Regulation of Inflammatory Responses of PDLCs by E3s

Recent research has shown that tripartite motif‐containing protein 31 (TRIM31), an E3 ubiquitin ligase of TRIMs, plays crucial roles in the development of various pathological conditions, including inflammatory diseases, bone diseases, viral infections and cancer progression [50]. Notably, a study confirmed that TRIM31 inhibited activation of the NLRP3 inflammasome in PDLCs, subsequently alleviating diabetic periodontitis [51]. In an in vitro model of diabetic periodontitis, advanced glycation end products (AGEs), oxidative derivatives resulting from diabetic hyperglycemia, led to a time‐dependent decrease in both the gene expression and protein levels of TRIM31 in PDLCs. Further mechanistic studies revealed that TRIM31 overexpression enhanced NLRP10 ubiquitination in PDLCs, a process that was reversed by the protease inhibitor MG132. Therefore, TRIM31 inhibited NLRP10 by promoting proteasomal degradation. NLRP10 also enhanced the activity of the NLRP3 inflammasome, thereby enhancing inflammatory responses [52]. Crucially, a previous study revealed that under physiological conditions, TRIM31 interacted with NLRP3 and induced its k48‐linked ubiquitination, subsequently mediating its degradation [51]. Taken together, the inhibition of TRIM31 by AGEs amplified the protein expression of NLRP10 and NLRP3, thereby intensifying periodontal inflammatory responses [52]. In summary, TRIM31 plays a protective role in counteracting AGE‐induced inflammatory responses, highlighting its potential as a promising therapeutic target in diabetic periodontitis.

### 3.2 Role of E3s in Cells Related to Periodontal Bone Metabolism

#### 3.2.1 Regulation of Bone Formation by Osteoblasts via E3s

F‐box protein 11 (FBXO11) serves as a crucial component of the F‐box protein family of E3s and exerts a profound influence on diverse physiological processes, including bone development, immune‐inflammatory responses and tumorigenesis [53]. Research showed that FBXO11 promoted osteoblast function by inhibiting the activity of Snail1, a repressor of osteogenic processes in osteoblasts [54]. Consistently, another study revealed that FBXO11 deficiency enhanced Snail1 protein levels in osteoblasts and consequently suppressed bone formation [55]. Further investigation is imperative to comprehend the regulatory functions of FBXO11 in inflammatory bone resorption within periodontal tissues and uncover the specific underlying mechanisms.

#### 3.2.2 Regulation of Bone Destruction of Osteoclasts by E3s

Nrf2 is an important antioxidant enzyme that regulates cellular oxidative stress and mitigates lipopolysaccharide‐mediated RANKL‐dependent bone destruction [56]. A previous study revealed that CUL3 modulated osteoclast function by ubiquitinating Nrf2, consequently promoting periodontal inflammatory bone resorption [41]. Another study also confirmed that CUL3 interacted with the substrate adaptor Keap1 to form the Keap1‐CUL3‐E3 complex, which facilitated the ubiquitination and subsequent degradation of Nrf2. Subsequently, the Keap1‐CUL3‐E3 complex enhanced ROS production by diminishing cellular antioxidant enzyme expression and subsequently provoked osteoclast differentiation and periodontal bone destruction [57]. Hence, targeting the Keap1‐CUL3‐E3 complex serves as a therapeutic strategy for managing periodontal bone loss. Given the essential regulatory role of CUL3 in periodontitis, CUL3 may represent a potential therapeutic target for periodontitis.

#### 3.2.3 Regulation of Infectious Osteolysis of Osteocytes by E3s

Osteocytes, the most prevalent and long‐lived cells within the bone matrix, serve as pivotal regulators of bone remodelling. They exercise their crucial roles through endocrine regulation and the modulation of calcium and phosphate metabolism [58]. Studies have revealed that specific E3s play crucial roles in modulating osteocytes [59, 60]. PDZ and LIM Domain 2 (PDLIM2) is a nuclear ubiquitin E3 ligase that protected osteocytes from inflammatory damage [60]. A recent investigation revealed that lentiviral overexpression of PDLIM2 suppressed gene expression and protein levels of RANKL in osteocytes, thereby inhibiting infectious osteolysis. Mechanistically, lentiviral overexpression of PDLIM2 enhanced the k48‐linked ubiquitination of STAT3, a vital contributor to bone resorption. Consistently, PDLIM2 deficiency resulted in increased STAT3 protein levels and elevated RANKL mRNA and protein levels, consequently restraining infectious osteolysis in osteocytes. Moreover, this study explored the upstream factors that regulated PDLIM2 expression. The findings revealed that activation of the MYD88 pathway, a central adapter protein in the downstream signalling cascades involving NF‐κB and JAK‐STAT3, suppressed both mRNA and protein levels of PDLIM2 in osteocytes. Consequently, this suppression hindered the ub‐independent degradation of STAT3, resulting in elevated STAT3 levels. As a result, increased STAT3 levels elevated RANKL expression and induced inflammatory osteolysis [59]. Regarding the protective role of PDLIM2, targeted activation of PDLIM2 in osteocytes may offer a promising therapeutic approach for periodontitis.

F‐box and leucine‐rich repeat protein 19 (FBXL19), a member of the Skp1‐Cullin‐F‐box family of E3s, plays crucial roles in essential biological processes, including cell proliferation, migration and differentiation [61]. A study confirmed that the lentiviral overexpression of FBXL19 diminished both the protein and mRNA levels of RANKL in osteocytes, subsequently suppressing inflammatory responses. Intricate mechanisms revealed that FBXL19 inhibited RANKL production in osteocytes by increasing k48‐linked ubiquitination of cAMP‐response element binding protein (CREB), a critical transcription factor for RANKL. Furthermore, *P. gingivalis* ‐mediated activation of MYD88 decreased the protein levels of FBXL19 and CREB, consequently enhancing RANKL transcription in osteocytes and aggravating bone resorption [59]. Therefore, targeted activation of FBXL19 may represent a potentially efficacious approach for preventing bone resorption in periodontitis.

### 3.3 Role of E3s in Immune Cells

#### 3.3.1 Regulation of Inflammatory Responses of PBMCs by E3s

PBMCs represent a diverse immune cell population in the peripheral blood and are crucial for periodontitis pathogenesis due to their role as the sole source of monocytes in periodontal supportive tissues. PBMCs significantly contribute to immune and inflammatory responses when exposed to *P. gingivalis* . Dysfunctional PBMCs predispose individuals to destructive immune responses, leading to periodontitis. A previous study showed that during periodontitis, PBMCs were recruited to the bone and undergone differentiation into active bone‐resorbing osteoclasts, thus providing evidence that PBMCs play a vital role in periodontitis progression [62]. This section reviews the mechanistic pathways involving E3s in the regulation of PBMCs and their clinical implications.

Neural precursor cells express developmentally downregulated 4 ligase (Nedd4‐2), a member of the Nedd4 family of E3s, which functions as a ub‐protein ligase to regulate the internalisation and turnover of various membrane proteins [63]. A study showed that Nedd4‐2 inhibited inflammatory responses by PBMCs and subsequently attenuated periodontitis. Researchers found that Nedd4‐2 deficiency using small interfering RNA (siRNA) significantly reduced pro‐inflammatory cytokine levels in *P. gingivalis* ‐stimulated PBMCs. Further investigation revealed that siRNA‐mediated silencing of Nedd4‐2 in *P. gingivalis* ‐stimulated PBMCs resulted in increased protein expression of Wnt3a, an anti‐inflammatory regulator that plays a significant role in controlling inflammation and bone metabolism in periodontitis. Mechanistically, Nedd4‐2 mediated k48‐linked ubiquitination of Wnt3a. This study further explored the upstream regulators of Nedd4‐2 and demonstrated that Janus Kinase 3 (JAK3) amplified Wnt3a signalling by phospho‐inactivating Nedd4‐2, thereby suppressing the infiltration of inflammatory cells in *P. gingivalis* ‐induced mice with periodontitis [64]. In conclusion, targeting the JAK3‐Nedd4‐2‐Wnt3a pathway may be a novel strategy for modulating periodontal inflammation.

#### 3.3.2 Regulation of Inflammatory Responses of Macrophages by E3s

TRAF2 and TRAF3 are members of the TRAF family of E3s, characterised by a RING finger domain and a zinc‐binding motif at the *N*‐terminus. TRAF2 is identified as an NF‐κB signalling activation regulator in cellular responses [65]. TRAF3 is expressed by activated macrophages and regulates various receptor‐mediated signalling pathways [66]. A study confirmed that in macrophages, TRAF2 phosphorylation led to TRAF3 degradation, promoting NF‐κB activity and thereby intensifying inflammatory responses. These findings indicated that TRAF2/3‐NF‐κB signalling plays a critical role in regulating inflammatory immune responses. In addition, the research revealed that TRAF2/3 was regulated by the upstream factor serum/glucocorticoid‐regulated kinase 1 (SGK1). Further investigation demonstrated that in response to *P. gingivalis* challenge, MG‐132, a ubiquitination‐associated proteasome inhibitor, abrogated the ability of SGKl deficiency to increase TRAF2 phosphorylation and decrease TRAF2/3 expression, suggesting that SGKl protected TRAF3 against degradation, possibly by inhibiting its ubiquitination. These findings further indicated that SGK1 suppression by the specific inhibitor EMD63868 resulted in elevated TRAF2 phosphorylation, thereby triggering TRAF3 ubiquitination‐independent degradation, activating the NF‐κB pathway and subsequently exacerbating periodontal inflammatory bone loss. In conclusion, the study identified an SGK1‐TRAF2/3‐NF‐κB signalling axis that is indispensable for the modulation of periodontitis progression. This finding suggests that the UPS components may serve as early therapeutic targets in periodontitis [67].

GSDMD, a key executor that triggers pyroptosis, serves as an attractive checkpoint in host defences against inflammatory and autoimmune diseases, such as periodontitis [68]. Synoviolin plays a crucial role in the recognition, ubiquitination and transport of proteins, and is thus involved in the endoplasmic reticulum‐associated protein degradation pathway. A study further demonstrated that compared with wild‐type mice, mice with synoviolin deficiency in myeloid cells presented aggravated periodontal tissue destruction with upregulated interleukin‐1β (IL‐1β) and IL‐18. Mechanistically, synoviolin deficiency led to decreased GSDMD ubiquitination and elevated GSDMD protein levels, subsequently aggravating inflammatory responses. Overall, synoviolin suppressed inflammasome activation and periodontitis by promoting GSDMD ubiquitination, suggesting its potential as a therapeutic target for managing periodontitis [69].

## 4 Regulatory Role of Dubs in Periodontitis

**FIGURE 3:**
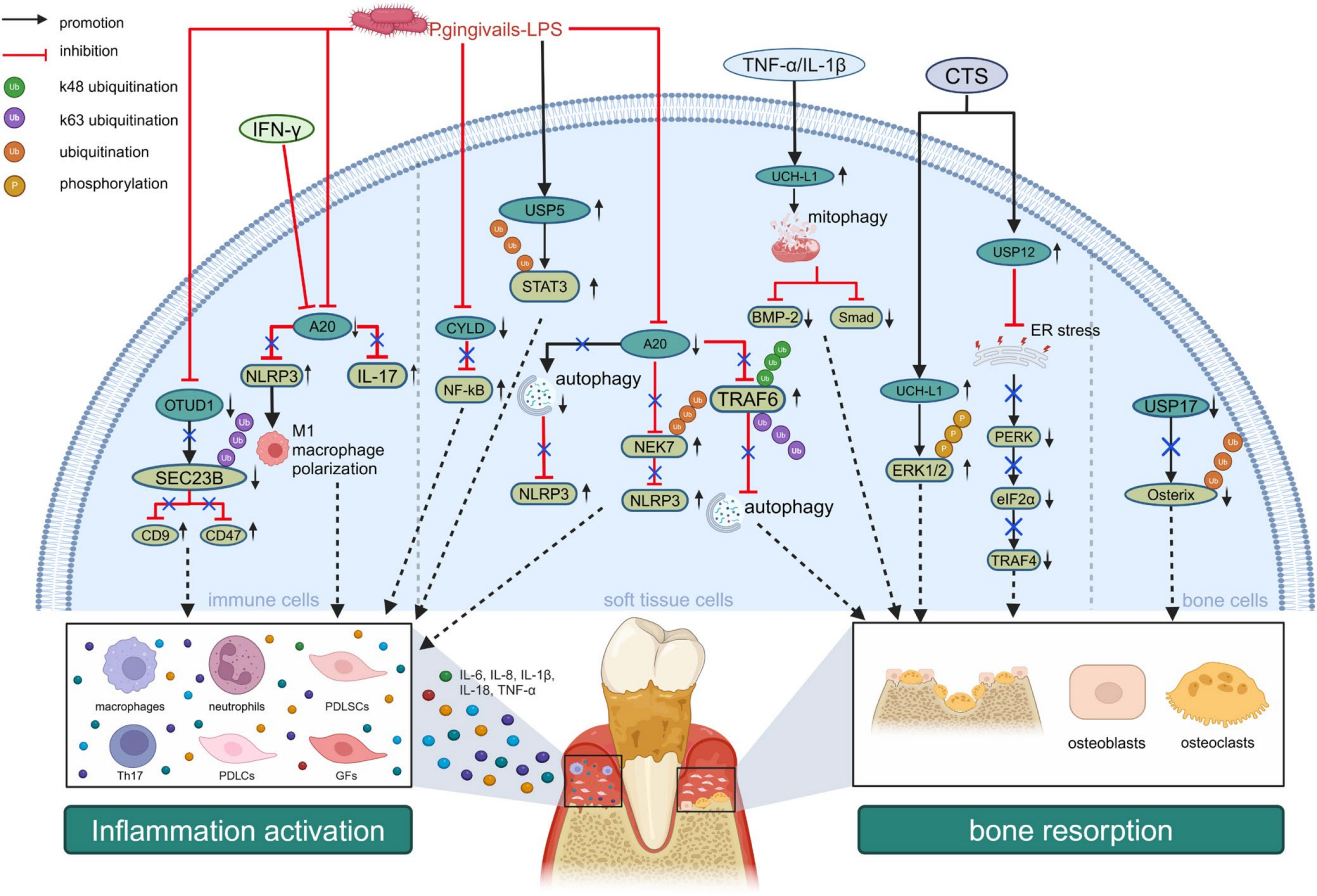
Effects of DUBs on periodontal inflammation and bone resorption. UCH‐L1 induced bone resorption by downregulating the mitophagy‐dependent BMP2/Smad signalling pathway and activating the ERK1/2 pathway. USP5 induced inflammation by deubiquitinating STAT3. USP12 escalated inflammation and endoplasmic reticulum stress by modulating the PERK/eIF2α/ATF4 signalling pathway under CTS. A20 suppressed bone resorption by deubiquitinating TRAF6. A20 inhibited inflammation through ubiquitination of NEK7 and deubiquitination of NLRP3 and IL‐17. CYLD negatively regulated p65/NF‐κB signalling triggered by P.g‐LPS. USP17 promoted osteoblast differentiation by stabilising Osterix. USP34 promoted bone formation by increasing Smad1 levels and deubiquitinating RUNX2. OTUD1 inhibited inflammation by deubiquitinating SEC23B.

DUB families, such as USPs, OTUs and UCHs, have been identified as key players in modulating periodontitis. This section provides an overview of recent research on the involvement of DUBs in regulating these periodontitis‐related cells and the underlying mechanisms involved (Figure 3).

### 4.1 Role of DUBs in Cells Related to Periodontal Soft Tissues

#### 4.1.1 Regulation of Osteogenesis and Inflammatory Responses of PDLSCs by DUBs

Ubiquitin C‐terminal hydrolases (UCHs), a subfamily of DUBs, are comprised of four members: UCH‐L1, UCH‐L3, UCHL5/UCH37 and BRCA1‐associated protein‐1 [70]. All UCH enzymes have a conserved catalytic domain (UCH domain) comprising approximately 230 amino acids [71]. Recently, considerable attention has been directed towards the role of UCH family members in regulating periodontitis.

A recent study confirmed the crucial role of UCH‐L1 in aggravating periodontitis, as reflected by increased mRNA and protein levels of UCH‐L1 in PDLSCs exposed to inflammatory cytokines and in patients with periodontitis compared with healthy controls. This research also revealed that UCH‐L1 knockdown enhanced ALP activity, calcium deposition and the mRNA expression of *RUNX2* and osteopontin by PDLSCs. Furthermore, the use of LDN57444, a specific UCH‐L1 inhibitor, enhanced the osteogenesis of PDLSCs exposed to tumour necrosis factor alpha (TNF‐α) or IL‐1β, suggesting a destructive role of UCH‐L1 in periodontitis progression. Moreover, an in vivo study consistently revealed that LDN57444 enhanced alveolar bone regeneration in mice with periodontitis. Mechanistically, osteogenesis suppression by UCH‐L1 was counteracted by LDN193189, an inhibitor of the bone morphogenetic protein 2/Smad (BMP2/Smad) signalling pathway, indicating that UCH‐L1 suppression promoted osteogenic differentiation through the BMP2/Smad signalling pathway [72]. Therefore, targeted suppression of UCH‐L1 represents a promising strategy for promoting alveolar bone regeneration in periodontal diseases.

Extensive research has investigated the involvement of the USP family, specifically ubiquitin‐specific protease 5 (USP5) and ubiquitin‐specific protease 12 (USP12), in modulating processes including periodontal inflammatory responses, osteoclastogenesis and osteoblastogenesis [56]. USPs modulated the production of pro‐inflammatory cytokines during periodontitis progression. Moreover, USPs modulated various osteogenic and osteoclastic signalling pathways, such as EGFR‐MAPK, NF‐κB/p65 and bone morphogenetic protein (BMP)/transforming growth factor β (TGF‐β) pathways [73]. Consequently, targeted USP intervention represents a promising therapeutic approach for periodontitis.

Research has revealed that compared to healthy controls, patients with chronic periodontitis exhibited elevated USP5 protein levels in both the gingival sulcus fluid and gingival tissues. Furthermore, a positive association has been established between USP5 overexpression and enhanced production of pro‐inflammatory cytokines, including TNF‐α, IL‐6 and IL‐1β in PDLSCs, suggesting that USP5 triggered inflammation and consequently aggravated periodontitis [74]. Furthermore, the downregulation of USP5 through siRNA transfection and application of the DUBs inhibitor, WP1130, decreased the protein expression of pro‐inflammatory cytokines by PDLSCs. Mechanistically, the knockdown of USP5 prevented the phosphorylation and activation of STAT3, effectively inhibiting LPS‐induced inflammatory responses in PDLSCs, indicating that targeted suppression of USP5 provides advantageous outcomes in mitigating periodontitis [74].

#### 4.1.2 Regulation of Osteogenesis and Inflammatory Responses of PDLCs by DUBs

USP12, a cysteine hydrolase DUB, is a key regulator of osteogenic differentiation of PDLCs stimulated by cyclic tension stress (CTS). One study demonstrated increased protein levels of USP12 in PDLCs subjected to CTS and on the tension side of the molar teeth during orthodontic tooth movement (OTM) in mice. Mechanistically, USP12 escalated inflammation and endoplasmic reticulum stress of PDLCs by modulating the protein kinase R‐like endoplasmic reticulum kinase/eukaryotic initiation factor 2α/activating transcription factor 4 (PERK/eIF2α/ATF4) signalling pathway under CTS [75]. In conclusion, USP12 inhibition is a protective strategy for periodontitis treatment.

A recent study also revealed that mechanical force induced UCH‐L1 expression by PDLCs during OTM. Notably, the findings demonstrated that knocking‐down UCH‐L1 using siRNA reduced the RANKL/OPG ratio in PDLCs, effectively inhibiting osteoclast differentiation. In vivo results further validated that LDN57444, a specific UCH‐L1 inhibitor, suppressed both OTM and osteoclast activity in rats. Furthermore, the results confirmed that mechanistically, activated UCH‐L1 in PDLCs promoted osteoclast differentiation via the ERK1/2 pathway, thereby facilitating OTM. Thus, UCH‐L1 plays a pivotal role in regulating OTM [76]. Investigating the regulatory functions of UCH‐L1 will deepen our understanding of OTM in patients with periodontitis.

Furthermore, OTUs have been implicated in human diseases such as cancer, neurodegeneration and viral infection [77]. Research has revealed that OTUs primarily regulated the release of pro‐inflammatory cytokines and osteogenic differentiation of PDLCs, thereby modulating periodontitis progression [16]. Recent research identified that tumour necrosis factor alpha‐induced protein 3 (TNFAIP3), also known as A20, structurally consisting of seven C‐terminal zinc finger domains and an *N*‐terminal ovarian tumour domain, acted as a negative regulator of the NF‐κB pathway and inhibited apoptosis of PDLCs [78, 79]. In response to P.g‐LPS and nicotine, the overexpression of A20 promoted autophagy, which in turn decreased pyroptosis and alleviated inflammation in PDLCs [80]. Another study demonstrated that A20 exerted protective effects on PDLCs by hindering osteoclastogenesis and reducing autophagy under hypoxic conditions. Mechanistically, A20 overexpression mitigated hypoxia‐induced osteoclastogenesis by suppressing TRAF6‐dependent autophagy and NF‐κB nuclear translocation, leading to decreased TNF‐α and IL‐1β levels, and reduced bone resorption. Notably, A20 suppressed autophagy and osteoclastogenesis in PDLCs under hypoxic conditions by regulating the k48‐linked and k63‐linked ubiquitination of TRAF6, consequently attenuating periodontitis progression [81]. Given the key regulatory role of A20 in inflammation and bone resorption, A20 agonists are promising drugs for the treatment of periodontitis.

#### 4.1.3 Regulation of Inflammatory Responses of GFs by DUBs

Gingival tissues serve as the primary defence against pathogen invasion in periodontitis. GFs, the predominant component of gingival tissue, serve as sentinel cells that modulate periodontal inflammatory responses to oral pathogens [82, 83].

Cylinderomatosis (CYLD) is a member of the USP family of DUBs. Previous studies revealed that CYLD regulated diverse cellular processes including immune responses, inflammation, cell cycle progression and osteoclastogenesis [84, 85]. Research further showed a significant decrease in CYLD protein levels in the inflamed gingival tissue of mice with periodontitis compared to healthy controls, suggesting a potential regulatory role of CYLD in periodontitis [86]. Furthermore, in GFs stimulated by P.g‐LPS, suppression of CYLD by siRNA significantly upregulated the mRNA levels of pro‐inflammatory cytokines, enhanced p65 phosphorylation and activated NF‐κB signalling. In addition, CYLD negatively regulated NF‐κB activation by deconjugating its k63‐linked ubiquitin chains [87]. These results showed that CYLD suppressed the p65/NF‐κB signalling pathway and subsequently alleviated periodontal inflammation [86]. Therefore, the targeted activation of CYLD may serve as a promising therapeutic strategy.

Recent studies have clarified various downstream factors regulated by A20. However, the upstream factors influencing A20 protein levels and activity remain largely unknown. A study confirmed that N6‐adenosine‐methyltransferase‐like 3 (METTL3), a well‐known RNA methyltransferase and a crucial regulator of inflammatory immune cells, was an upstream modulator of A20 [88]. A recent study further revealed that METTL3‐mediated N6‐methyladenosine (m^6^A) promoted A20 degradation. Mechanistically, siRNA‐mediated METTL3 deficiency resulted in the accumulation of A20, which facilitated the ubiquitination of NIMA‐related kinase 7 (NEK7) and subsequently suppressed the NLRP3 inflammasome assembly. Furthermore, researchers have found that coptisine chloride, a novel METTL3 inhibitor, dose‐dependently downregulated NEK7 protein expression. Additionally, coptisine chloride administration significantly ameliorated inflammatory periodontal bone loss in mice with periodontitis, suggesting that it is a promising therapeutic drug against periodontitis. This study revealed a novel pathogenic mechanism involving METTL3‐A20‐NEK7 in periodontitis [89]. Taken together, these results suggest that A20 plays important roles in the progression of periodontitis and may serve as a potential therapeutic target.

### 4.2 Role of DUBs in Cells Related to Periodontal Bone Metabolism

Periodontal bone homeostasis involves a delicate dynamic balance between bone resorption and formation [90]. The regulatory roles of DUBs in bone regeneration, specifically in the modulation of osteoblasts and osteoclasts, have garnered increasing attention.

#### 4.2.1 Regulation of Inflammatory Responses and Bone Formation of Osteoblasts by DUBs

Osteoblasts are the principal cells involved in periodontal bone formation [91]. They synthesise osteoid matrix and regulate the mineralisation process [91, 92]. Previous studies highlighted the pivotal role of USPs in regulating osteoblast differentiation [93]. One study revealed that USP17 promoted osteoblast differentiation by stabilising osterix, an osteoblast‐specific transcription factor [94]. Moreover, another study revealed that A20 derived from skeletal stem cells alleviated rat osteoarthritis by inhibiting the necroptosis of subchondral osteoblasts [95]. Additionally, conditional osteoblast knockout of the deubiquitinase OTUB1 resulted in low bone mass and poor bone strength due to defects in osteogenic differentiation and mineralization [96]. Although numerous reports have elucidated the involvement of USPs in the regulation of osteoblasts, further comprehensive investigations are warranted to clarify the specific roles of USPs in osteoblasts during periodontitis.

#### 4.2.2 Regulation of Bone Loss of Osteoclasts by DUBs

Osteoclasts are giant, multinucleated, bone‐resorbing cells that are central to osteolytic diseases, including periodontitis. Various pathological factors, including TNF‐α, IL‐1β, IL‐6, IL‐17 and RANKL, modulate the differentiation, activation and survival of osteoclasts [92]. Furthermore, inhibition of osteoclastogenesis has been proven to ameliorate periodontal alveolar bone loss [97]. Therefore, it is imperative to elucidate the regulatory mechanisms by which DUBs modulate osteoclast activity in order to develop effective therapeutic strategies for periodontitis.

A previous study demonstrated that A20 inhibited osteoclastogenesis by suppressing NF‐κB and TRAF6 pathways in PDLCs [81]. Furthermore, in mice with periodontitis, overexpression of A20 in gingival tissues using adeno‐associated viral (AAV) vectors decreased periodontal bone resorption [98]. Other research demonstrated that CYLD, a predominant DUB that regulated NF‐κB, inhibited osteoclastogenesis and consequently ameliorated alveolar bone loss. Further investigation revealed that compared with wild‐type mice, CYLD knockout mice with periodontitis manifested an increased number of osteoclasts, reduced osteogenesis and subsequently aggravated alveolar bone loss [99]. Therefore, the targeted regulation of CYLD presents a burgeoning avenue for periodontitis treatment. Nonetheless, research on DUBs modulating osteoclastogenesis remains limited. Future studies should delve deeper into the potential functions and mechanisms of action of DUBs in osteoclasts, thereby opening new therapeutic opportunities for the treatment of periodontitis.

### 4.3 Role of DUBs in Immune Cells

#### 4.3.1 Regulation of Inflammatory Responses of Macrophages by DUBs

Macrophages are key innate immune cells in periodontal tissues that significantly affect periodontitis progression through chemotaxis, phagocytosis and release of inflammatory cytokines. Macrophages are typically categorised into pro‐inflammatory M1 and anti‐inflammatory M2 types [100]. A study revealed that in human monocytic‐leukaemia cells (THP‐1), A20 inhibited the inflammasome pathway induced by P.g‐LPS and interferon, thereby reducing the NLRP3‐mediated polarisation of M1 macrophages. Concurrently, the deletion of A20 using siRNA in human macrophage‐like cells and THP‐1 increased the secretion of inflammatory cytokines such as IL‐6 and TNF‐α. In conclusion, A20 attenuated periodontitis progression by suppressing NLRP3‐mediated M1 macrophage polarisation, suggesting the potential of A20 as a novel therapeutic target for periodontitis management [98].

#### 4.3.2 Regulation of Inflammatory Responses of Neutrophils by DUBs

A recent study indicated that neutrophils that infiltrate tissues, known as tissue‐infiltrating neutrophils (Tins), play a crucial role in the development of periodontal disease by enhancing cytokine production and facilitating migration. Furthermore, OTU domain‐containing protein 1 (OTUD1) diminished inflammatory responses, migration and polarisation capabilities of Tins. Clinical investigations further revealed a notable increase in both the mRNA and protein levels of OTUD1 in the gingival tissues of patients with periodontitis compared to healthy controls. Furthermore, OTUD1 knockout mice displayed increased periodontal bone resorption and inflammation. Mechanistically, OTUD1 suppressed the process of protein endoplasmic reticulum‐to‐Golgi trafficking by removing k63‐linked ubiquitination of Sec23 homologue B (SEC23B), a coat protein II complex component. These findings validated the OTUD1/SEC23B axis as a potential therapeutic target for periodontitis. Furthermore, blocking protein transport using Brefeldin A (BFA) curbed the recruitment of OTUD1‐deficient Tins and attenuated inflammation‐induced alveolar bone destruction [101]. These results thus revealed the role of OTUD1 in limiting the polarisation of neutrophils with a secretory phenotype and highlight the potential application of BFA in the treatment of periodontal inflammation.

#### 4.3.3 Regulation of Inflammatory Responses of Th17 by DUBs

T helper 17 cells (Th17) are a subset of pro‐inflammatory T helper cells defined by their production of interleukin 17 (IL‐17). The IL‐17 family, a relatively new family of cytokines, comprises six ligands (IL‐17A to IL‐17F) that bind to five receptor subtypes (IL‐17RA to IL‐17RE) and induce downstream signalling [102]. Previous in vivo and in vitro studies indicated that A20 binded to IL‐17 RA in fibroblasts to cease IL‐17A signalling pathways, and subsequently diminished protein levels of pro‐inflammatory cytokines [103, 104]. Furthermore, A20 hinderd Th17 recruitment and osteoclast activation by inhibiting the maturation of IL‐1β [103]. Nonetheless, numerous aspects of A20 and Th17 interactions, including the cellular mechanisms by which the ZnF7 motif in A20 restricts Th17 differentiation and the interplay between the IL‐17/IL‐23 axis and A20, remain unclear [103].

## 5 Regulation of Ups for Periodontitis Treatment: Limitations and Prospects

Bortezomib (BTZ), a potent proteasome inhibitor, suppresses the proteolytic activity of the 26S proteasome by binding to its catalytic core [105, 106, 107]. An in vivo study revealed that BTZ reduced the protein expression of inflammatory cytokines including TNF‐α, IL‐1β, IL‐6 and IL‐8, decreased the ratio of RANKL to osteoprotegerin (OPG), and thus hindered periodontal alveolar bone absorption in mice [108, 109]. Furthermore, previous research has demonstrated that BTZ directly inhibited RANKL‐ and P.g‐LPS‐dependent osteoclast differentiation in mice with periodontitis [110, 111]. These findings illustrate the anti‐inflammatory and anti‐osteoclastic properties of BTZ in combating periodontitis and suggest that BTZ represents a promising therapeutic agent for periodontitis. Although BTZ has received FDA approval for the treatment of multiple myeloma and mantle cell lymphoma [112], studies investigating its clinical efficacy against periodontitis are lacking. Furthermore, studies have confirmed that DI‐1548 and DI‐1859, potent and specific inhibitors of CUL3, activated the SHH/Gli1 and Nrf2 pathways, subsequently suppressing inflammation and inhibiting apoptosis of PDLSCs [42]. These findings underscore the potential therapeutic effects of targeted inhibitors of UPS enzymes, including BTZ, DI‐1548 and DI‐1859, in the treatment of periodontitis, which warrants further investigation.

In addition to the chemical inhibitors that regulate UPS enzymes, periodontal pathogens, particularly *P. gingivalis* , play crucial roles in modulating UPS enzymes. *P. gingivalis* produces proteolytic enzymes known as gingipains that degrade collagen, fibronectin and laminin, thereby disrupting tissue integrity and enhancing the pathological progression of periodontitis [113]. Moreover, P.g‐LPS, another key virulence factor of *P. gingivalis* , modulated multiple classical pathways, such as MYD88 [59], SGK1 [67] and JAK3 [64], subsequently altering the expression of E3s and DUBs, including CUL3 [41], synoviolin [69], OTUD1 [101], A20 [79], CYLD [99] and USP5 [74]. Notably, *Fusobacterium nucleatum* ( *F. nucleatum* ), another major bacterial pathogen in periodontitis, activated the NF‐κB and TLR4/MYD88 signalling pathways by regulating UPS enzymes, notably USP25 and MDM2 [114, 115, 116]. Collectively, *P. gingivalis* and *F. nucleatum* play crucial roles in the modulation of UPS enzymes. Consequently, targeted modulation of UPS enzymes through chemical antimicrobial treatments may emerge as a novel therapeutic approach for periodontitis, which warrants further investigation. In summary, chemical interventions, including inhibitors and antimicrobial strategies designed to modulate the UPS may provide innovative therapeutic strategies for periodontitis.

Meanwhile, targeted regulation of the UPS through genetic intervention presents another promising therapeutic approach for the management of periodontitis. The remarkable safety and precise targeting capabilities of AAV vectors underscore the preclinical and clinical applications of gene replacement, silencing and editing, suggesting promising therapeutic strategies for the management of various clinical diseases [117]. A specific study demonstrated that AAV‐facilitated overexpression of A20 effectively mitigated inflammatory responses and reduced periodontal bone loss in mice, highlighting the potential efficacy of AAV in periodontal therapies [98]. However, the application of AAV in modulating DUBs for the treatment of periodontitis is currently limited. Additionally, AAV‐mediated overexpression of the E3 ubiquitin ligase Hsp70 interacting protein mitigated the cognitive and pathological phenotypes of mice with Alzheimer [118]. However, no comprehensive studies have explored the impact of AAV‐mediated E3 gene alterations on periodontitis progression, indicating the need for further research.

The modulation of E3s and DUBs through chemical and genetic interventions represents potential and effective strategies for periodontitis treatment. Nevertheless, further clinical trials and in‐depth studies related to the targeted modulation of E3s and DUBs remain limited and require further exploration.

## 6 Conclusion

This review underscores the essential regulatory role of the UPS in the pathogenesis of periodontitis (Tables 1 and 2). E3s and DUBs interact with a variety of substrates that collectively play an indispensable role in influencing the pathological progression of periodontitis [16]. Moreover, the modulation of E3s and DUBs through chemical and genetic interventions represents a potentially effective strategy for periodontitis treatment. Owing to the complex mechanisms of the UPS in periodontitis, more comprehensive research is required to elucidate the potential targeted regulation of the UPS in periodontitis and pave the way for targeted therapies for periodontitis.

**TABLE 1:** Summary of studies on E3s in periodontitis.

Species	E3s	Stimulation	Cell types	Targets	Function	Potential clinical significance	References
Human	TRIM16	P.g‐LPS	PDLSCs	PICOT and CHIP ↓	Osteogenic differentiation (+)	Activation of TRIM16 is a promising therapeutic strategy for periodontitis	[46, 49]
Human	TRIM31	AGEs	PDLCs	NLRP10 and NLRP3 ↓	Periodontal inflammatory response (−)	Activation of TRIM31 is a promising therapeutic strategy for periodontitis	[51, 52]
Human	CUL3	P.g‐LPS	PDLSCs	Nrf2 and SHH/Gli ↓	Periodontal inflammatory responses (+)	DI‐1548 and DI‐1859, selective CUL3 inhibitors, are promising therapeutic agents for periodontitis	[41, 42]
Mouse	CUL3	RANKL	Osteoclasts	Nrf2 ↓	Osteoclast differentiation (+)	Inhibition of Keap1‐CUL3‐E3 complex is a promising therapeutic strategy for periodontitis	[57]
Mouse	FBXL19	P.g‐LPS	Osteocytes	CREB ↓	Periodontal inflammatory response (−)	Activation of FBXL19 is a promising therapeutic strategy for periodontitis	[59]
Mouse	PDLIM2	P.g‐LPS	Osteocytes	STAT3 ↓	Periodontal inflammatory response (−)	Activation of PDLIM2 is a promising therapeutic strategy for periodontitis	[59]
Mouse	TRAF2/3	P.g‐LPS	Macrophages	NF‐κB ↓	Periodontal inflammatory response (−)	Activation of SGK1‐TRAF2/3–NF‐κB is a promising therapeutic strategy for periodontitis	[67]
Mouse	Synoviolin	P.g‐LPS	Macrophages	GSDMD ↓	Periodontal inflammatory response (−)	Activation of Synoviolin is a promising therapeutic strategy for periodontitis	[69]
Mouse	Nedd4‐2	P.g‐LPS	PBMCs	Wnt3a ↓	Periodontal inflammatory response (−)	Activation of JAK3‐Nedd4‐2‐Wnt3a is a promising therapeutic strategy for periodontitis	[64]

*Note*: ↑, Upregulation; ↓, Downregulation. (+), Potentiation; (−), Attenuation.

**TABLE 2:** Summary of studies on DUBs in periodontitis.

Species	DUBs	Stimulation	Cell types	Targets	Function	Potential clinical significance	References
Human	A20	P.g‐LPS	PDLCs	TRAF6 and NF‐κB ↑	Periodontal inflammation and bone resorption (−)	Activation of A20 is a promising therapeutic strategy for periodontitis	[78, 79, 80, 81]
Mouse	A20	P.g‐LPS	GFs	NEK7 ↑	Periodontal inflammation and bone resorption (−)	Coptisine Chloride, a selective inhibitor of METTL3, represents a promising therapeutic agent for periodontitis	[89]
Human and Mouse	A20	IFN‐γ and P.g‐LPS	Macrophages	NLRP3 ↑	Periodontal inflammatory response (−)	Activation of A20 is a promising therapeutic strategy for periodontitis	[98]
Human and Mouse	A20	IFN‐γ and P.g‐LPS	Th17 cells	IL‐17 ↑	Periodontal inflammatory response (−)	Activation of A20 is a promising therapeutic strategy for periodontitis	[102, 103, 104]
Mouse	UCH‐L1	TNF‐α/IL‐1β	PDLSCs	BMP‐2 and Smad ↓	Osteogenic differentiation (+)	LDN193189, the BMP2/ Smad signalling pathway inhibitor, is a promising therapeutic agent for periodontitis	[72]
Human and Mouse	UCH‐L1	CTS	PDLCs	ERK1/2 ↑	Osteoclast differentiation (+)	Inhibition of UCH‐L1 is a promising therapeutic strategy for OTM in patients with periodontitis	[76]
Mouse	CYLD	P.g‐LPS	GFs	NF‐κB ↑	Periodontal inflammatory responses (+)	Subquinocin, a specific CYLD inhibitor, represents a promising therapeutic agent for periodontitis	[86]
Mouse	CYLD	P.g‐LPS	Osteoclasts	NF‐κB and Wnt/β‐catenin ↑	Osteoclast differentiation (+)	Inhibition of CYLD is a promising therapeutic strategy for periodontitis	[99]
Human	USP5	P.g‐LPS	PDLSCs	STAT3 ↑	Periodontal inflammatory response (+)	WP1130, a USP5 inhibitor, is a promising therapeutic agent for periodontitis	[74]
Human and Mouse	USP12	CTS	PDLCs	PERK/eIF2α/ATF4 pathway ↓	Osteogenic differentiation (−)	Inhibition of USP12 is a promising therapeutic strategy for OTM in patients with periodontitis	[75]
Human	USP17	BMP4	Osteoblast	Osterix ↑	Osteoblast differentiation (+)	Activation of USP17 is a promising therapeutic strategy for periodontitis	[94]
Human and Mouse	OTUD1	P.g‐LPS	Neutrophils	SEC23B ↑	Periodontal inflammatory response (−)	Activation of OTUD1 is a promising therapeutic strategy for periodontitis	[101]

*Note*: ↑, Upregulation; ↓, Downregulation. (+), Potentiation; (−), Attenuation.

## Author Contributions

**Yilin Ma:** writing – original draft. **Ruiwei Jia:** writing – original draft. **Shuhong Chen:** writing – original draft. **Jun Ma:** formal analysis. **Lei Yin:** formal analysis. **Xingbei Pan:** formal analysis. **Yunuo He:** formal analysis. **Tong Wu:** project administration. **Zheyu Zhao:** project administration. **Shengzhuang Wu:** writing – review and editing. **Huining Wang:** writing – review and editing. **Guang Liang:** writing – review and editing. **Shengbin Huang:** conceptualization, writing – review and editing. **Xiaoyu Sun:** conceptualization, writing – review and editing.

## Conflicts of Interest

The authors declare no conflicts of interest.

## Data Availability

The data that support the findings of this study are available from the corresponding author upon reasonable request.
